# Effects of Double-Stage Annealing Parameters on Tensile Mechanical Properties of Initial Aging Deformed GH4169 Superalloy

**DOI:** 10.3390/ma14154339

**Published:** 2021-08-03

**Authors:** Guanqiang Wang, Mingsong Chen, Yongcheng Lin, Yumin Lou, Hongbin Li, Yanyong Ma, Zonghuai Zou, Quan Chen, Yuchi Xia

**Affiliations:** 1 School of Mechanical and Electrical Engineering, Central South University, Changsha 410083, China; wgq001@csu.edu.cn (G.W.); yjsyzx@ncst.edu.cn (H.L.); zndxmyy01@163.com (Y.M.); 15573271714@163.com (Z.Z.); 203711004@csu.edu.cn (Q.C.); xiayuchi@csu.edu.cn (Y.X.); 2 State Key Laboratory of High Performance Complex Manufacturing, Central South University, Changsha 410083, China; 3 Light Alloy Research Institute, Central South University, Changsha 410083, China; 4 Zheneng Technology Research Institute Co., Ltd., Hangzhou 310026, China; CQ2013118525@163.com; 5 College of Metallurgies and Energy, North China Science and Technologies University, Tangshan 063009, China

**Keywords:** GH4169 superalloy, annealing treatment, tensile test, mechanical properties

## Abstract

This study takes large size samples after hot-upsetting as research objects and aims to investigate the optimization double-stage annealing parameters for improving the mechanical properties of hot-upsetting samples. The double-stage annealing treatments and uniaxial tensile tests for hot-upsetting GH4169 superalloy were finished firstly. Then, the fracture mode was also studied. The results show that the strength of hot-upsetting GH4169 superalloy can be improved by the double-stage annealing treatment, but the effect of annealing parameters on the elongation of GH4169 alloy at high temperature and room temperature is not significant. The fracture mode of annealed samples at high-temperature and room-temperature tensile tests is a mixture of shear fracture and quasi-cleavage fracture while that of hot-upsetting sample is a shear fracture. The macroscopic expressions for the two fracture modes belong to ductile fracture. Moreover, it is also found that the improvement of strength by the double-stage annealing treatment is greater than the single-stage annealing treatment. This is because the homogeneity of grains plays an important role in the improvement of strength for GH4169 superalloy when the average grain size is similar. Based on a comprehensive consideration, the optimal annealing route is determined as 900 °C × 9–12 h(water cooling) + 980 °C × 60 min(water cooling).

## 1. Introduction

Ni-based superalloys are widely used for the manufacture of key components in the aerospace and energy industries due to their good comprehensive mechanical properties [1,2]. Generally, these components are often made by hot forming. To get high-quality forgings, the thermal deformation behavior of materials should be studied deeply [3,4,5]. Presently, some studies have been carried out on the hot deformation behaviors of some Ni-based superalloys, such as Inconel 625 [6], Alloy 617 [7,8], Monel400 Ni-Cu alloy [9], C276 [10], Nimonic 80A [11,12], GH378 [13], Incoloy 901 [14], and GH4169 (Inconel 718) [15,16,17]. Among them, GH4169 superalloy is the most used one in the aerospace industry. For GH4169 superalloy, γ″ (Ni_3_Nb) and γ′ (Ni_3_Al) are the main precipitation strengthening phases [18]. δ phase (Ni_3_Nb), an equilibrium phase of the metastable γ″ phase, has pronounced effects on material properties [19], the hot deformation [20] and microstructural evolution [21]. Lin et al. [20,21] studied the flow behaviors of GH4169 superalloy firstly and then established the phenomenological and physically based constitutive models. Wen et al. [22,23] and He et al. [24] established the hot processing maps of the superalloy. Some scholars studied the microstructure evolution by cellular automaton (CA) simulation [25,26,27]. However, since the poor thermal conductivity of GH4169 superalloy and the narrow range of forming parameters, complete dynamic recrystallization is difficult to occur in the forming process, which leads to the occurrence of the mixed grains [28,29]. The existence of mixed grains will reduce the mechanical properties. To get high-quality forgings, they must be eliminated.

The annealing treatment could be an effective method to eliminate the mixed grain due to the recrystallization behavior during annealing. However, few studies have been done on the recrystallization behavior during annealing treatment. In the authors’ previous work [30], it was found that the large deformed grains have been substituted by recrystallization grains after a single annealing treatment because of the occurrence of static recrystallization (SRX). However, some large coarse recrystallized grains are produced. This is because the high driving pressure and the weak pinning effect of the δ phase promote the quick growth of dynamic recrystallized (DRX) grain nuclei [31]. In order to avoid the appearance of coarse recrystallized grains, Chen et al. [32] designed the double-stage annealing treatment and found that the double-stage annealing treatment has a better effect on refining grain and improving the homogeneity of microstructure compared with the single annealing treatment.

The microstructure of forgings determines their mechanical properties [33,34]. Uniform and fine grains are beneficial to the mechanical properties of forgings [35,36]. In the authors’ previous work [32], it was found that the double-stage annealing treatment is an effective way to eliminate the mixed grains of deformed GH4169 superalloy, but there are few studies on the improvement of mechanical properties during the double-stage annealing treatment up to now. Moreover, to simplify the experimental process, the small-size sample (ϕ10 mm × 15 mm) deformed on a Gleeble-3500 is selected as the study object in the author’s previous study [32]. Due to the volume effect of metal components, the obtained optimized annealing parameters can only provide a reference on the design of double-stage annealing treatments for large forgings. Hence, this study takes the large size samples (ϕ100 mm × 140 mm) after hot-upsetting as research objects and aims to investigate the optimized annealing parameters for improving the mechanical properties of hot-upsetting samples. The samples of GH4169 superalloy were forged firstly and then annealed by diverse annealing treatments. After that, for these annealed samples, the tensile test was implemented, and the fracture mode of GH4169 superalloy was also investigated.

## 2. Materials and Experiments

**Figure 1:**
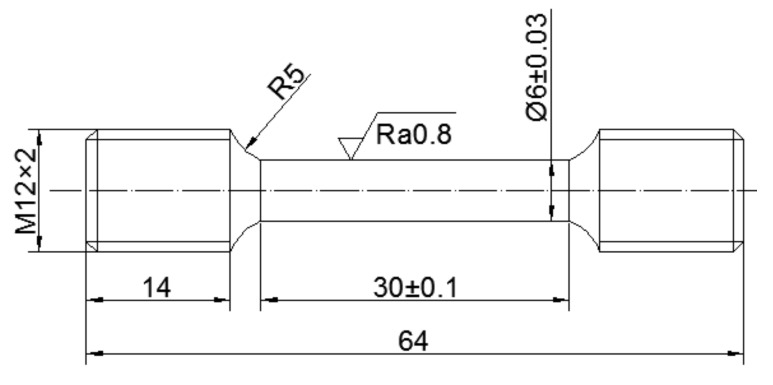
Geometry dimension of uniaxial tensile samples (unit: mm).

**Figure 2:**
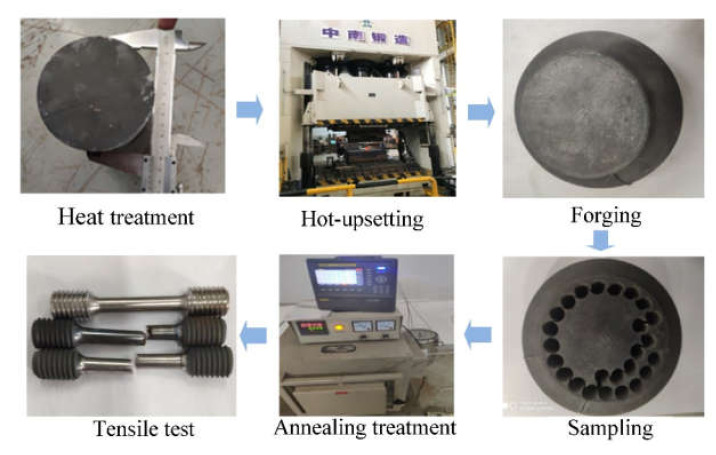
Experimental procedure diagram for the whole test.

A commercial GH4169 superalloy of which chemical compositions (wt. %) is 52.82Ni-18.96Cr-5.23Nb-3.01Mo-1.00Ti-0.59Al-0.01Co-0.03C-(bal.)Fe was used in this study. To obtain the deformed microstructure, there was a hot-upsetting. The size of the specimen for hot-upsetting is ϕ100 mm × 140 mm. Before hot-upsetting, the sample was solution-treated (T = 1040 °C, t = 0.75 h). Then, an aging treatment (T = 900 °C, t = 24 h) for precipitating δ phase was done. For the hot-upsetting, the deformation temperature was 950 °C, the strain rate was 0.01 s^−1^ and the true strain was 0.69. After hot-upsetting, the uniaxial tensile samples were machined from the deformed specimen according to ISO 6982-2 [37]. The geometry dimension of the uniaxial tensile sample is displayed in Figure 1. Before uniaxial tensile tests, all the tensile samples were treated by annealing treatments illustrated in Table 1. The first-stage annealing (FSA) and second-stage annealing (SSA) aim to precipitate the δ phase and promote recrystallization, respectively. Moreover, the strengthening heat treatment (per AMS 5596) was added for all uniaxial tensile specimens to precipitate the strengthen γ′′ and γ′ phases [38,39]. In addition, to investigate the difference of tensile behavior at high temperature and room temperature, cases 3 and 9 in addition to 4 and 10 were annealed in the same condition of annealing but deformed under different tensile tests. The selection of annealed parameters is based on the authors’ previous study [30,32]. After that, the high temperature and room temperature uniaxial tensile tests were conducted on a MTS-GWT2105 machine, and the detailed uniaxial tensile experimental schemes are shown in Table 2. The flow diagram of the whole test is demonstrated in Figure 2.

To investigate the relationship between microstructure and mechanical properties during double-stage annealing treatment, Optical Microscope (OM), Electron Back-Scattered Diffraction (EBSD), and Scanning Electron Microscopy (SEM) technologies were applied for the microstructure observation. The detailed preparation methods for OM, SEM and EBSD can be found in References [40,41]. The analysis of microstructure evolution based on the OM and EBSD technologies has been introduced in the authors’ previous study [32]. Thus, in this study, only SEM technology in the TESCAN MIRA3 LMU was employed to observe the fracture morphologies of tensile specimens.

## 3. Results and Analysis

Generally, during the uniaxial tensile test, the gauge section of the sample is regarded as uniformly elongated before fracture. Therefore, in the gauge section, the area and change rate of all sections are the same at any time. Moreover, the engineering stress ($\sigma_{E}$) and engineering strain ($\varepsilon_{E}$) can be expressed as

(1) $$\sigma_{E}=\frac{F}{A_{0}},\varepsilon_{E}=\frac{\Delta L}{L_{0}}$$

where F is the applied external load, $A_{0}$ is the cross-sectional area of the gauge section, $L_{0}$ is the length of the gauge section, and ΔL is the elongation of the gauge section in the tensile test.

### 3.1. Effects of Double-Stage Annealing Parameters on High-Temperature Tensile Behaviors

#### 3.1.1. Effect of FSA Time on High-Temperature Tensile Behaviors

(1) Tensile mechanical properties

**Figure 3:**
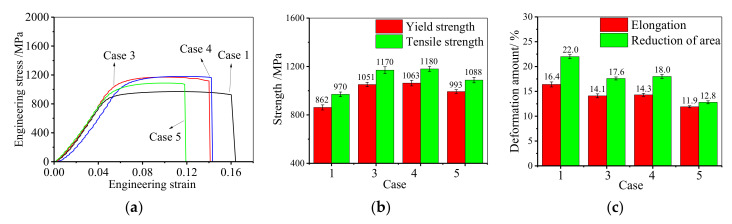
Effect of FSA time on tensile mechanical properties: (**a**) engineering stress–engineering strain curve; (**b**) strength; (**c**) elongation to fracture.

Figure 3 shows the influence of the FSA time on mechanical properties. From Figure 3a, it can be found that there is no obvious elastic-plastic transition period, which is consistent with the normal behavior reported in References [42,43,44]. Thus, the offset yield strength corresponding to 0.2% plastic strain (called yield strength) is used. In the plastic deformation period, because of the continuous proliferation, accumulation and interaction of dislocation, the strain hardening effect of GH4169 superalloy becomes obvious. Compared to the hot-upsetting sample, the strain hardening of the annealed samples is stronger. Figure 3b illustrates the effect of FSA time on the strength of GH4169 superalloy. Compared to the hot-upsetting sample (case 1), the strength of the annealed samples (cases 3, 4, and 5) significantly increases. This is attributed to the three main strengthening mechanisms for Ni-based superalloy [45]: (a) solid solution strengthening (the local nonuniformity in the lattice resulted from the solute atoms (i.e., alloying element) makes plastic deformation more difficult by impeding dislocation motion through stress fields [46]); (b) precipitation strengthening (the precipitation strengthening comes out when the second phase hinders the movement of dislocations to harden the materials [47]); (c) grain-boundary strengthening, i.e., fine-grain strengthening (the grain boundaries also act as pinning points impeding further dislocation propagation to increase the strength [48]). On the one hand, there are more recrystallized grains in the annealed samples, which increases the number of grain boundaries. During tensile deformation, the resistance to slip is larger, and then the strength improves. On the other hand, there are some δ phases in the sample, and it results in a decrease in the fraction of strengthening γ′′ phase after the strengthening heat treatment [49]. Compared to the hot-upsetting sample (case 1), the fraction of δ phase for annealed samples (cases 3, 4, and 5) is larger. It means that the precipitation strengthening is weaker. However, the strength of the annealed samples is larger than that of the hot-upsetting sample. This illustrates that the strength of the studied alloy after annealing greatly increases because the fine-grain strengthening plays a leading role in the combined effect of fine-grain strengthening and precipitation strengthening.

From Figure 3b, it can also be noticed that the strength increases firstly and then decreases when the FSA time is increased from 9 h to 24 h. This is because the large deformed grains are gradually replaced by SRX grains, and the SRX grain size is still small when the FSA time is less than 12 h [32]. Thus, both the yield and tensile strengths slightly increase when the FSA time climbs from 9 h to 12 h. However, since the further growth of SRX grains, the average grain size increases when the FSA time ascends to 24 h. Therefore, the effect of fine-grain strengthening becomes weak. However, the fractions of δ phases after aging at 900 °C for 12 h and 24 h are similar because the precipitation of δ phase has reached saturation after aging at 900 °C for 12 h [32]. Namely, the effect of precipitation strengthening is similar for cases 4 and 5. Consequently, both the yield and tensile strengths significantly decrease as the FSA time further increases to 24 h.

Figure 3c shows the influence of the FSA time on the elongation and reduction of area. It can be found that the elongation to fracture of the annealed samples is smaller compared to the hot-upsetting sample. This is because there are more grain boundaries in annealed samples due to the occurrence of static recrystallization. In the tensile deformation, the dislocation will accumulate near the grain boundaries, and it will hinder the movement of dislocation. Therefore, for annealed samples, the tensile deformation is more difficult, which leads to a decrease in the elongation and reduction of area. Moreover, since the δ phase promotes the crack propagation during tensile deformation [19], the tensile specimen is easier to fracture with the increase of FSA times. Thus, the elongation to fracture slightly drops from 14.1% to 11.9% when the FSA time climbs from 9 h to 24 h.

In short, the FSA time of double-stage annealing treatment mainly affects the mechanical properties of deformed samples by improving their strength. The optimal range of FSA time is 9 h–12 h because both the strength and elongation are higher.

(2) Tensile fracture mode

**Figure 4:**
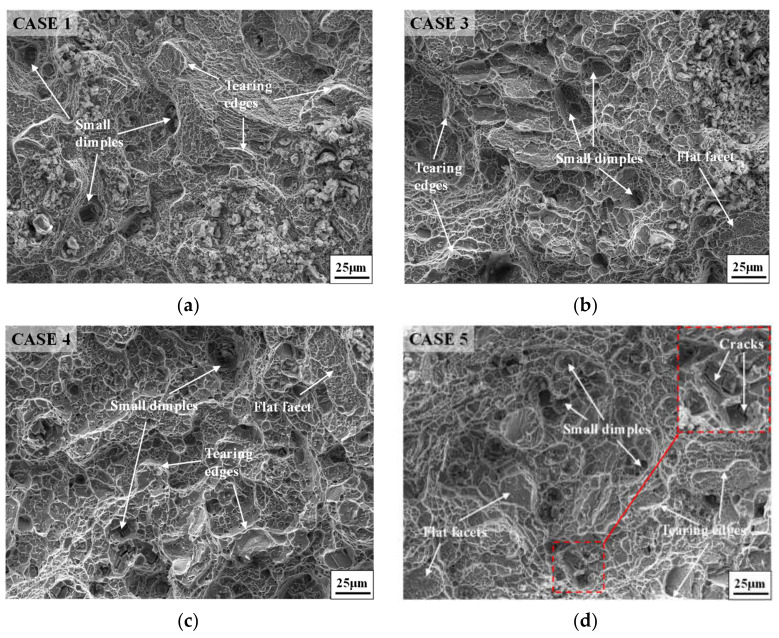
Fracture morphologies of (**a**) hot-upsetting sample; and annealed samples with different FSA times: (**b**) 9 h; (**c**) 12 h; (**d**) 24 h.

Figure 4 shows the fracture morphologies of annealed samples with different FSA times and the hot-upsetting sample. Obviously, the fracture morphologies of annealed samples with different FSA times and the hot-upsetting sample have the same characteristics. It can be observed that lots of tearing edges generate at the fracture surface, and many small dimples distribute inside the fracture surface. Generally, the large dimension of dimple reflects the high plasticity of material, and large numbers of dimples and tearing edges represent the occurrence of shear fracture [50,51]. This indicates that the high-temperature fracture modes of annealed samples with different FSA times and the hot-upsetting sample are the shear fracture. Moreover, for annealed samples with different FSA times, there are a few flat facets (i.e., cleavage planes) in which some small tearing edges locate compared to Figure 4a, as shown in Figure 4b–d. This illustrates that the high-temperature fracture modes of annealed samples with different FSA times contain quasi-cleavage fracture [52,53]. Therefore, for annealed samples with different FSA times, the high-temperature fracture mode is a mixture of shear fracture and quasi-cleavage fracture. The occurrence of quasi-cleavage fracture leads to the reduction of plasticity [54,55], so the elongation of annealed sample is decreased. However, owing to the appearance of many dimples and some cleavage planes, the fracture mode presents a ductile fracture macroscopically. Furthermore, from Figure 4d, it is also found that some small cracks come out, resulting in the occurrence of fracture and reduction of plasticity. This phenomenon is responsible for the small drop of elongation for case 5.

#### 3.1.2. Effect of SSA Time on High-Temperature Tensile Behavior

(1) Tensile mechanical properties

**Figure 5:**
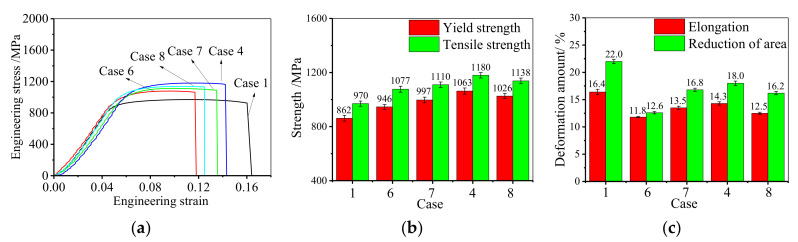
Effect of SSA time on high-temperature tensile behavior: (**a**) engineering stress–engineering strain curve; (**b**) strength; (**c**) elongation to fracture.

Figure 5 demonstrates the role of SSA time on mechanical properties. Similarly, the uniaxial tensile curve is mainly divided into two stages, and there is no obvious elastic-plastic transition stage, as depicted in Figure 5a. During the uniaxial tensile deformation, all the samples yield quickly. Compared to the hot-upsetting sample, the strain hardening of the annealed sample is stronger. Figure 5b illustrates the influence of SSA time on the strength of GH4169 superalloy. Compared to the hot-upsetting sample, the strengths of annealed samples significantly increase. This attributes to that the fine-grain strengthening is significantly enhanced by recrystallization during the SSA. Moreover, the strength increases firstly and then decreases when the SSA time is increased from 10 min to 90 min (cases 4, 6, 7, and 8). Based on statistical analysis, when the SSA time is increased to 90 min, the yield strength increases from 862 MPa to 1063 MPa and then decreases to 1026 MPa, and the tensile strength increases from 970 MPa to 1180 MPa and then decreases to 1138 MPa. This is consistent with the evolution of microstructure, and the detailed reasons are listed as follows. In the primary stage of SSA, the average grain size decreases, and the microstructure becomes more uniform with the rise of SSA time due to the occurrence of recrystallization. Therefore, the fine-grain strengthening is stronger and stronger with the rise of SSA time, which leads to an increase in strength. However, there is full recrystallization when the SSA time reaches to 60 min [32]. Only grain growth occurs in the subsequent annealing treatment, which weakens the fine-grain strengthening. Thus, the strength decreases in the subsequent annealing treatment. Meanwhile, for the sample aged at 900 °C for 12 h, the effect of the precipitation strengthening is similar after annealing at 980 °C for 60 min because the dissolution of δ phase has reached a balance after annealing at 980 °C for 60 min (the fraction of δ phase is 3.7%) [32]. Under the combined effects of both the precipitation strengthening and fine-grain strengthening, the strength increases first and then decreases. Figure 5c demonstrates the effect of SSA time on the elongation and reduction of area. It can be noticed that the elongations to fracture of the annealed samples drop compared to the hot-upsetting sample. This is because large numbers of new recrystallized grain boundaries hinder the motion of dislocation during tensile deformation for annealed samples. Moreover, since the δ phase promotes the crack propagation during tensile deformation, the elongation to fracture increases until the SSA time reaches 60 min. After that, the value of elongation to fracture drops a little with the rise of SSA time. Hence, the SSA time of double-stage annealing treatment mainly affects the mechanical properties of deformed samples by improving their strength. The optimal parameter of FSA time is 60 min because both the strength and elongation are higher.

(2) Tensile fracture mode

**Figure 6:**
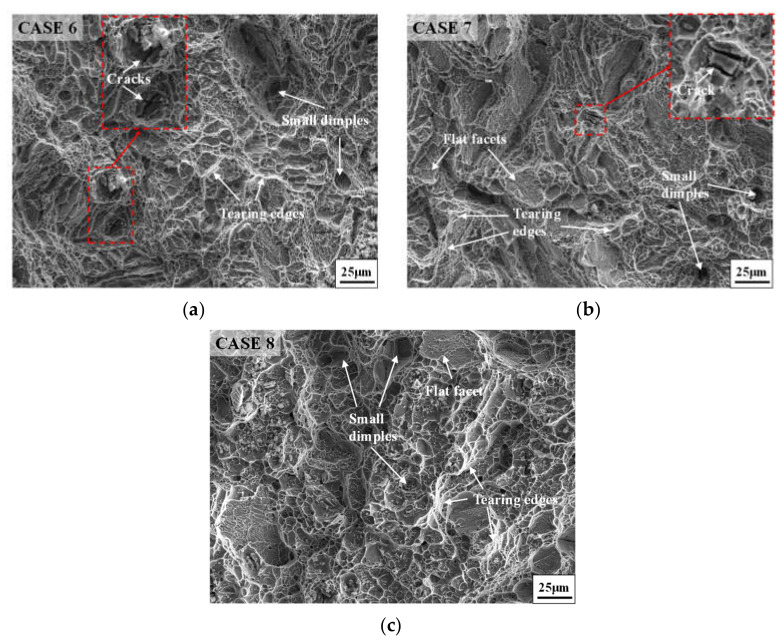
Fracture morphologies of annealed samples with different SSA times: (**a**) 10 min; (**b**) 30 min; (**c**) 90 min.

Figure 6 shows the fracture morphologies of annealed samples with different SSA times. From Figure 4c and Figure 6, it can be found that the fracture characteristics of the studied superalloy with different SSA times are almost the same. A large number of tearing edges generate at the fracture surface, and many small dimples distribute inside the fracture surface. Moreover, it can be noticed that a few cleavage planes locate at the fracture surface, and some small tearing edges generate on those cleavage planes. These phenomena demonstrate that the high-temperature fracture mode for annealed samples with different SSA times is a mixture of shear fracture and quasi-cleavage fracture. Furthermore, the fracture mode presents a ductile fracture macroscopically due to the existence of large numbers of dimples and few cleavage planes. This indicates that the effect of SSA time on the high-temperature fracture mode of GH4169 superalloy is not obvious. In addition, the small cracks observed in Figure 6a–c are responsible for the small drop of elongation for cases 6, 7, and 8.

Based on the above results, the double-stage annealing treatments mainly affect the high-temperature tensile mechanical properties of the deformed sample by improving the strength. The optimal double-stage annealing route is 900 °C × 9–12 h(water cooling) + 980 °C × 60 min(water cooling). The variation role of high-temperature tensile mechanical properties for annealed samples is strongly related to the microstructure evolution during the double-stage annealing. Furthermore, the high-temperature fracture mode of deformed GH4169 superalloy after double-stage annealing treatments is a mixture of shear fracture and quasi-cleavage fracture and presents a ductile fracture macroscopically. Therefore, it is necessary to study the mechanical properties and fracture mode at room temperature of deformed GH4169 superalloy after double-stage annealing treatments with optimal parameters.

### 3.2. Comparison of High-Temperature and Room-Temperature Tensile Behavior

**Figure 7:**
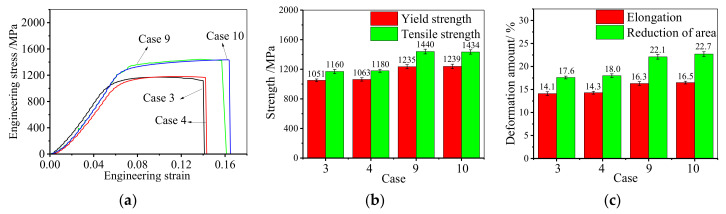
Comparisons of tensile mechanical properties at room-temperature and high-temperature: (**a**) engineering stress–engineering strain curve; (**b**) strength; (**c**) elongation to fracture.

The comparisons of mechanical properties at room temperature and high temperature are shown in Figure 7. From Figure 7a, apparently, the strength of the studied alloy at the room-temperature tensile test is larger than that at the high-temperature tensile test. Both the strengths of the annealed samples at room temperature and high temperature have been significantly improved compared with that of the hot-upsetting sample. This indicates that adopting a suitable double-stage annealing treatment after deformation is an effective way to improve the strength of deformed GH4169 superalloy. The comparisons of the strength and elongation to fracture at room temperature and high temperature are illustrated in Figure 7b,c. It can be observed that the strength and elongation to fracture of cases 3 and 4 are similar, so do cases 9 and 10. This is because the deformed samples that undergo the double-stage annealing treatments of 900 °C × 9–12 h(water cooling) + 980 °C × 60 min(water cooling) have the similar microstructure. Therefore, the effects of fine-grain strengthening and precipitation strengthening are similar. Furthermore, it is noteworthy that the strength and the elongation at room temperature are larger than that at high temperature. This is attributed to the softening of grain boundary and the efficient movement of dislocations during high-temperature deformation. On the one hand, the softening of grain boundary weakens the resistance capability of grain boundary on dislocation slip [50]. On the other hand, the nucleation of microvoids on the δ phase is easier to take place due to the quick movement of dislocations [19]. Hence, both the strength and elongation at high temperature are smaller than that at room temperature.

**Figure 8:**
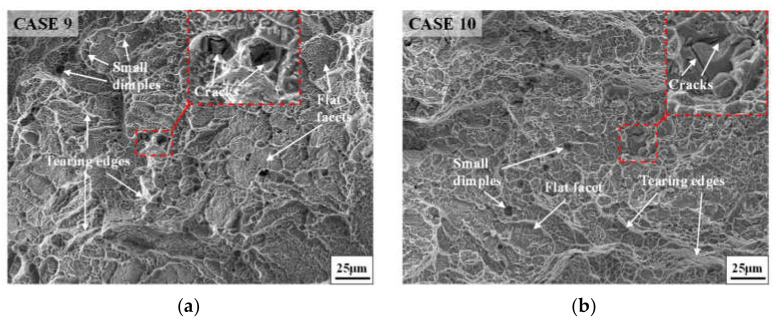
Fracture morphologies of annealed samples at room temperature tensile: (**a**) case 9; (**b**) case 10.

Figure 8 shows the fracture morphologies of the studied superalloy after tensile tests at room temperature. From Figure 4b,c and Figure 8, it can be noticed that the fracture morphologies of annealed samples after tensile tests at room temperature and high temperature have the same characteristics. A large number of tearing edges generate at the fracture surface, and many small dimples distribute inside the fracture surface. Moreover, a few cleavage planes in which some small tearing edges exist can be observed. Therefore, the fracture mode of annealed samples after tensile tests at room temperature is also a mixture of shear fracture and quasi-cleavage fracture. Moreover, the fracture mode also presents a ductile fracture macroscopically due to the appearance of many dimples and some cleavage planes. These phenomena indicate that both the fracture modes at room temperature and high temperature are similar.

## 4. Discussion

**Figure 9:**
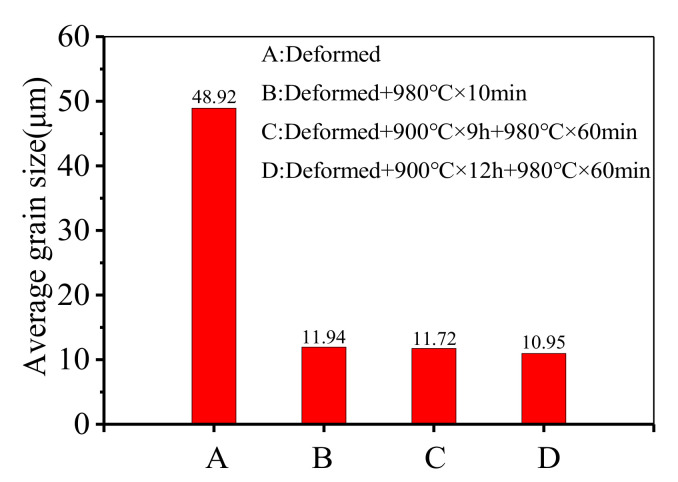
The average grain size after different annealing treatments.

In the authors’ previous works [30,32], it can be found that the annealing treatments, including the single-stage annealing treatment and the double-stage annealing treatment, can refine the deformed grains. The average grain size after different annealing treatments is shown in Figure 9. It can be found that the average grain size of the deformed GH4169 superalloy after annealing is similar. Therefore, it is necessary to study the effects of different annealing treatment methods on the improvement of mechanical properties.

**Figure 10:**
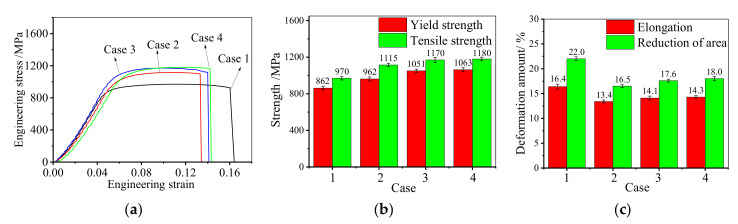
Effect of two types of annealing treatments on high-temperature tensile mechanical properties: (**a**) engineering stress–engineering strain curve; (**b**) strength; (**c**) elongation to fracture.

The effects of single-stage and double-stage annealing treatments on the high-temperature tensile behavior are shown in Figure 10. From Figure 10a, it is noteworthy that the strengths of samples after annealing treatments have been significantly increased compared with that of the hot-upsetting sample. It is because that the annealing treatments can induce full recrystallization, which makes the grains fine and uniform. However, there is some difference in the role of single-stage and double-stage annealing treatments on the high-temperature tensile behavior. The comparison for the influence of two types of annealing treatments on the yield strength and tensile strength are shown in Figure 10b. It is noticed that the yield strength increases from 862 MPa to 962 MPa after single-stage annealing treatment. However, after double-stage annealing treatments of 900 °C × 9 h(water cooling) + 980 °C × 60 min(water cooling) and 900 °C × 12 h(water cooling) + 980 °C × 60 min(water cooling), the yield strength of the deformed sample increases from 862 MPa to 1051 MPa and 1063 MPa. This implies that the improvement in strength by double-stage annealing treatments is greater than that of single-stage annealing treatments. The reasons are as follows. Although the single-stage annealing treatments can refine grain, the grains are still heterogeneous [31,32]. The relatively large recrystallized grains come from the quick growth of DRX nuclei and grains, while the very fine grains result from SRX nuclei. Therefore, the final grains are still heterogeneous. However, the microstructure becomes fine and homogeneous after double-stage annealing treatments with the appropriate annealing parameters [32].

**Figure 11:**
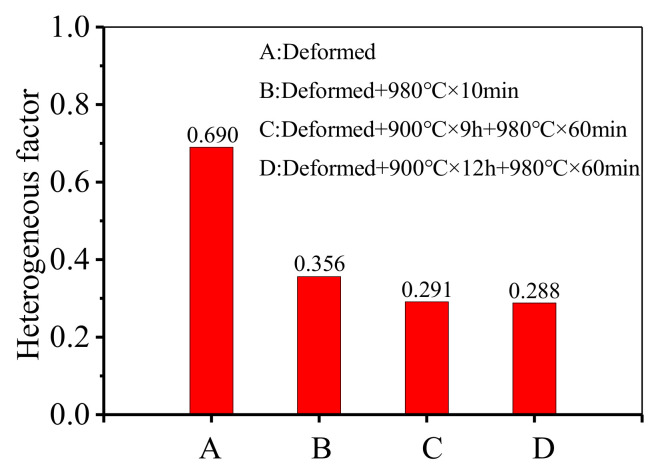
Heterogeneous factor (HF) after different annealing treatments.

The heterogeneous factor (HF) [56] of the deformed GH4169 superalloy after annealing is shown in Figure 11. According to the definition of HF, the larger the HF is, the more heterogeneous the grain is [56]. It can be found that the values of HF for deformed samples after double-stage annealing treatments are smaller. This means that the grains of the deformed GH4169 superalloy after double-stage annealing treatments are more uniform. The phenomenon suggests that the homogeneity of grains plays a major role in the improvement of the tensile mechanical properties for GH4169 superalloy when the average grain size is similar.

The comparisons of the effects of two types of annealing treatments on the elongation and reduction of area are shown in Figure 10c. It can be found that the elongation to fracture of the annealed samples decreases compared to the hot-upsetting sample, and the difference in the elongation between the two types of annealing treatments is small. This implies that the effect of homogeneity on the elongation to fracture is not obvious.

**Figure 12:**
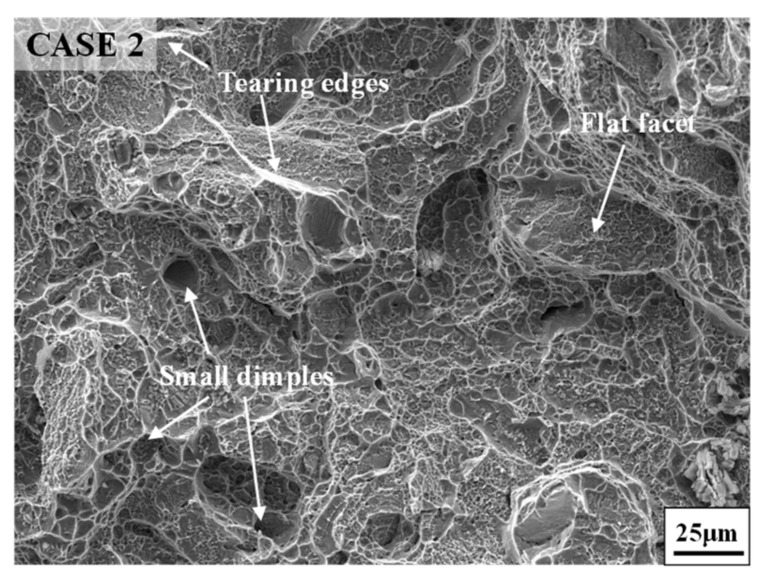
Fracture morphology of the studied superalloy with single annealing treatment.

Figure 12 displays the fracture morphology of the studied superalloy after single-stage annealing treatment. From Figure 4b,c and Figure 12, it can be noticed that the fracture morphologies of the studied superalloy with different annealing treatments are almost the same. The fracture mode of annealed samples after single-stage annealing treatment is also a mixture of shear fracture and quasi-cleavage fracture. Moreover, the fracture mode presents a ductile fracture macroscopically due to the appearance of many dimples and some cleavage planes. Hence, the fracture mode of annealed samples after single-stage annealing treatment and double-stage annealing treatment is similar.

## 5. Conclusions

The annealing treatments and tensile tests have been finished to study the effect of double-stage annealing parameters on tensile mechanical properties for deformed GH4169 superalloy. The fracture mode has also been studied. The conclusions are as follows:

(1) The strength of deformed GH4169 superalloy can be improved by annealing treatments. The double-stage annealing treatment has a better effect on the improvement of the strength for deformed GH4169 superalloy compared to the single-stage annealing treatment. This attributes to that the homogeneity of grains plays a major role in the improvement of strength when the average grain size is similar.

(2) The effect of annealing parameters on the elongation at high temperature and room temperature is not significant. The fracture mode of annealed samples at high-temperature and room-temperature tensile tests is a mixture of shear fracture and quasi-cleavage fracture while that of hot-upsetting sample is a shear fracture. The macroscopic expressions for the two fracture modes belong to ductile fracture.

(3) The optimal double-stage annealing process is 900 °C × 9–12 h(water cooling) + 980 °C × 60 min(water cooling). The yield strength of deformed sample is increased from 862 MPa to 1051 MPa and 1063 MPa after the double-stage annealing treatments of 900 °C × 9 h(water cooling) + 980 °C × 60 min(water cooling) and 900 °C × 12 h(water cooling) + 980 °C × 60 min(water cooling), respectively. Moreover, the tensile strength also increases from 970 MPa to 1160 MPa and 1180 MPa, respectively.

## Figures and Tables

**Table 1:** Annealing treatments after hot-upsetting (WC represents water cooling while FC indicates the furnace cooling to 620 °C at 50 °C/h).

Case	Double-Stage Annealing Treatment		Strengthening Heat Treatment
	Annealing Parameters of First-Stage Annealing (FSA)	Annealing Parameters of Second-Stage Annealing (SSA)	
1	/	/	720 °C × 8 h(FC) + 620 °C × 8 h(WC)
2	/	980 °C × 10 min(WC) [30]	
3	900 °C × 9 h(WC)	980 °C × 60 min(WC)	
4	900 °C × 12 h(WC)	980 °C × 60 min(WC)	
5	900 °C × 24 h(WC)	980 °C × 60 min(WC)	
6	900 °C × 12 h(WC)	980 °C × 10 min(WC)	
7	900 °C × 12 h(WC)	980 °C × 30 min(WC)	
8	900 °C × 12 h(WC)	980 °C × 90 min(WC)	
9	900 °C × 9 h(WC)	980 °C × 60 min(WC)	
10	900 °C × 12 h(WC)	980 °C × 60 min(WC)	

**Table 2:** Tensile experimental schemes.

Case	Temperature/°C	Strain Rate/s^−1^
1–8	650 (High temperature)	0.001
9–10	20 (Room temperature)	

## Data Availability

The raw/processed data required to reproduce these findings cannot be shared at this time as the data also forms part of an ongoing study.

## References

[1] Geng P., Qin G., Ma H., Zhou J., Ma N. (2021). Linear friction welding of dissimilar Ni-based superalloys: Microstructure evolution and thermo-mechanical interaction. J. Mater. Res. Technol..

[2] Kong W., Wang Y., Yuan C., Zhang B. (2021). Microstructural evolution and stress rupture behaviour of a Ni-Based wrought superalloy during thermal exposure. Mater. Sci. Eng. A.

[3] Chen F., Wang H., Zhu H., Cui Z. (2019). Study on Dynamic Recrystallization Behaviors in a Hot-Deformed FB2 Ultra-supercritical Rotor Steel. Metallogr. Microstruct. Anal..

[4] Bobbili R., Madhu V. (2015). An investigation into hot deformation characteristics and processing maps of high-strength armor steel. J. Mater. Eng. Perform..

[5] Chen F., Wang H., Zhu H., Zhu H., Ren F., Cui Z. (2019). High-temperature deformation mechanisms and physical-based constitutive modeling of ultra-supercritical rotor steel. J. Manuf. Process..

[6] Liu X., Fan J., Zhang P., Xie J., Chen F., Liu D., Yuan R., Tang B., Kou H., Li J. (2021). Temperature dependence of deformation behavior, microstructure evolution and fracture mechanism of Inconel 625 superalloy. J. Alloy. Compd..

[7] Pradhan S.K., Mandal S., Athreya C.N., Babu K.A., De Boer B., Sarma V.S. (2017). Influence of processing parameters on dynamic recrystallization and the associated annealing twin boundary evolution in a nickel base superalloy. Mater. Sci. Eng. A.

[8] Prithiv T.S., Bhuyan P., Pradhan S.K., Sarma V.S., Mandal S. (2018). A critical evaluation on efficacy of recrystallization vs. strain induced boundary migration in achieving grain boundary engineered microstructure in a Ni-base superalloy. Acta Mater..

[9] Ebrahimi G.R., Momeni A., Ezatpour H.R., Jahazi M., Bocher P. (2019). Dynamic recrystallization in Monel400 Ni-Cu alloy: Mechanism and role of twinning. Mater. Sci. Eng. A.

[10] Zhang C., Zhang L., Shen W., Xu Q., Cui Y. (2017). The processing map and microstructure evolution of Ni-Cr-Mo-based C276 superalloy during hot compression. J. Alloy. Compd..

[11] Quan G.Z., Zhang L., Wang X. (2017). Evolution of grain refinement degree induced by dynamic recrystallization for Nimonic 80A during hot compression process and its FEM analysis. Vacuum.

[12] Quan G.Z., Pan J., Wang X., Zhang Z., Zhang L., Wang T. (2017). Correspondence between grain refinements and flow softening behaviors at Nimonic 80A superalloy under different strain rates, temperatures and strains. Mater. Sci. Eng. A.

[13] Ma W., Luo H., Yang X. (2020). The Effects of Grain Size and Twins Density on High Temperature Oxidation Behavior of Nickel-Based Superalloy GH738. Materials.

[14] Momeni A., Abbasi S.M., Morakabati M., Badri H., Wang X. (2014). Dynamic recrystallization behavior and constitutive analysis of Incoloy 901 under hot working condition. Mater. Sci. Eng. A.

[15] Chen F., Liu J., Ou H., Lu B., Cui Z., Long H. (2015). Flow characteristics and intrinsic workability of IN718 superalloy. Mater. Sci. Eng. A.

[16] Lin Y.C., Wen D., Deng J., Liu G., Chen J. (2014). Constitutive models for high-temperature flow behaviors of a Ni-based superalloy. Mater. Des..

[17] Lin Y.C., Chen X., Wen D., Chen M. (2014). A physically-based constitutive model for a typical nickel-based superalloy. Comput. Mater. Sci..

[18] Lin Y.C., Yang H., Li L. (2017). Effects of solutionizing cooling processing on γ (Ni3Nb) phase and work hardening characteristics of a Ni-Fe-Cr-base superalloy. Vacuum.

[19] Lin Y.C., Deng J., Jiang Y., Wen D., Liu G. (2014). Effects of initial δ phase on hot tensile deformation behaviors and fracture characteristics of a typical Ni-based superalloy. Mater. Sci. Eng. A.

[20] Wen D., Lin Y.C., Zhou Y. (2017). A new dynamic recrystallization kinetics model for a Nb containing Ni-Fe-Cr-base superalloy considering influences of initial δ phase. Vacuum.

[21] He D., Lin Y.C., Wang L., Wu Q., Zu Z., Cheng H. (2019). Influences of pre-precipitated δ phase on microstructures and hot compressive deformation features of a nickel-based superalloy. Vacuum.

[22] Wen D., Lin Y.C., Li H., Chen X., Deng J., Li L. (2014). Hot deformation behavior and processing map of a typical Ni-based superalloy. Mater. Sci. Eng. A.

[23] Wen D.X., Lin Y.C., Chen J., Deng J., Chen X.M., Zhang J.L., He M. (2015). Effects of initial aging time on processing map and microstructures of a nickel-based superalloy. Mater. Sci. Eng. A.

[24] He D., Lin Y.C., Chen M., Chen J., Wen D., Chen X. (2015). Effect of pre-treatment on hot deformation behavior and processing map of an aged nickel-based superalloy. J. Alloy. Compd..

[25] Zhu H., Chen F., Zhang H., Cui Z. (2020). Review on modeling and simulation of microstructure evolution during dynamic recrystallization using cellular automaton method. Sci. China Technol. Sci..

[26] Liu Y., Lin Y.C., Zhou Y. (2017). 2D cellular automaton simulation of hot deformation behavior in a Ni-based superalloy under varying thermal-mechanical conditions. Mater. Sci. Eng. A.

[27] Chen F., Zhu H., Chen W., Ou H., Cui Z. (2021). Multiscale modeling of discontinuous dynamic recrystallization during hot working by coupling multilevel cellular automaton and finite element method. Int. J. Plast..

[28] Chen M.S., Lin Y.C., Li K., Zhou Y. (2016). A new method to establish dynamic recrystallization kinetics model of a typical solution-treated Ni-based superalloy. Comput. Mater. Sci..

[29] Chen M.S., Li K., Lin Y.C., Yuan W. (2016). An improved kinetics model to describe dynamic recrystallization behavior under inconstant deformation conditions. J. Mater. Res..

[30] Chen M.S., Zou Z., Lin Y.C., Li H., Yuan W. (2018). Effects of annealing parameters on microstructural evolution of a typical nickel-based superalloy during annealing treatment. Mater. Charact..

[31] Chen M.S., Zou Z., Lin Y.C., Li H., Wang G. (2019). Formation mechanism of large grains inside annealed microstructure of GH4169 superalloy by cellular automation method. J. Mater. Sci. Technol..

[32] Chen M.S., Zou Z.H., Lin Y.C., Li H.B., Wang G.Q., Ma Y.Y. (2019). Microstructural evolution and grain refinement mechanisms of a Ni-based superalloy during a two-stage annealing treatment. Mater. Charact..

[33] Agnoli A., Bernacki M., Logé R., Franchet J., Laigo J., Bozzolo N. (2015). Selective growth of low stored energy grains during δ sub-solvus annealing in the Inconel 718 nickel-based superalloy. Metall. Mater. Trans. A.

[34] Guo C., Yu J., Liu J., Sun X., Zhou Y. (2021). Tensile Deformation and Fracture Behavior of Nickel-Based Superalloy DZ951G. Materials.

[35] Nasiri Z., Ghaemifar S., Naghizadeh M., Mirzadeh H. (2021). Thermal Mechanisms of Grain Refinement in Steels: A Review. Met. Mater. Int..

[36] Najafkhani F., Kheiri S., Pourbahari B., Mirzadeh H. (2021). Recent advances in the kinetics of normal/abnormal grain growth: A review. Arch. Civ. Mech. Eng..

[37] ISO (2011). ISO 6892-2, Metallic Materials–Tensile Testing–Part 2: Method of Test at Elevated Temperature.

[38] Zhang C., Yu L., Wang H. (2019). Kinetic Analysis for High-Temperature Coarsening of γ Phase in Ni-Based Superalloy GH4169. Materials.

[39] Zhao M., Zhao Z., Liu L., Luo G., Chen W. (2020). Influence of Heat Treatment on Cyclic Response of Nickel-Based Superalloy Inconel 718 up to Very-High Cycle Regime. Materials.

[40] Chen M.S., Wang G.Q., Li H., Lin Y.C., Zou Z., Ma Y., He D., Zeng W. (2019). Precipitation and dissolution behaviors of 6 phase inside a deformed nickel-based superalloy during annealing treatment. Appl. Phys. A.

[41] Wang G.Q., Li H., Chen M.S., Lin Y.C., Zeng W., Ma Y., Chen Q., Jiang Y. (2021). Effect of initial mixed grain microstructure state of deformed Ni-based superalloy on its refinement behavior during two-stage annealing treatment. Mater. Charact..

[42] Zhang H., Li C., Guo Q., Ma Z., Huang Y., Li H., Liu Y. (2018). Hot tensile behavior of cold-rolled Inconel 718 alloy at 650 °C: The role of δ phase. Mater. Sci. Eng. A.

[43] Ye N., Cheng M., Zhang S., Song H., Zhou H., Wang P. (2015). Effect of δ phase on mechanical properties of GH4169 alloy at room temperature. J. Iron Steel Res. Int..

[44] Zhu W., Zhao F., Yin S., Liu Y., Yang R. (2021). Effect of Tensile Deformation on Residual Stress of GH4169 Alloy. Materials.

[45] Zhang S., Lin X., Wang L., Yu X., Hu Y., Yang H., Lei L., Huang W. (2021). Strengthening mechanisms in selective laser-melted Inconel718 superalloy. Mater. Sci. Eng. A.

[46] Pelleg J. (2012). Mechanical Properties of Materials.

[47] Gladman T. (1999). Precipitation hardening in metals. Mater. Sci. Technol..

[48] Callister W.D. (2000). Fundamentals of Materials Science and Engineering.

[49] Wang G.Q., Chen M.S., Li H., Lin Y.C., Zeng W., Ma Y. (2021). Methods and mechanisms for uniformly refining deformed mixed and coarse grains inside a solution-treated Ni-based superalloy by two-stage heat treatment. J. Mater. Sci. Technol..

[50] Wen D., Gao C., Zheng Z., Wang K., Xiong Y., Wang J., Li J. (2021). Hot tensile behavior of a low-alloyed ultrahigh strength steel: Fracture mechanism and physically-based constitutive model. J. Mater. Res. Technol..

[51] Hasnaoui A., Van Swygenhoven H., Derlet P.M. (2003). Dimples on nanocrystalline fracture surfaces as evidence for shear plane formation. Science.

[52] Martin M.L., Fenske J.A., Liu G.S., Sofronis P., Robertson I.M. (2011). On the formation and nature of quasi-cleavage fracture surfaces in hydrogen embrittled steels. Acta Mater..

[53] Meyers M.A., Chawla K.K. (2008). Mechanical Behavior of Materials.

[54] Yoshinaka F., Sawaguchi T. (2020). Characterization of crystallographic fracture surfaces in Fe–33Mn–6Si alloy. Int. J. Fatigue.

[55] Zhang S., Wang Y., Zhu M., Zhang Z., Nie P., Li Z. (2020). Relationships among Charpy impact toughness, microstructure and fracture behavior in 10CrNi3MoV steel weld joint. Mater. Lett..

[56] Chen M.S., Wang G.Q., Li H., Lin Y., Zou Z., Ma Y. (2019). Annealing Treatment Methods and Mechanisms for Refining Mixed and Coarse Grains in a Solution Treatment Nickel-Based Superalloy. Adv. Eng. Mater..

